# Complex-Scene SAR Aircraft Recognition Combining Attention Mechanism and Inner Convolution Operator

**DOI:** 10.3390/s25154749

**Published:** 2025-08-01

**Authors:** Wansi Liu, Huan Wang, Jiapeng Duan, Lixiang Cao, Teng Feng, Xiaomin Tian

**Affiliations:** 1 School of Remote Sensing and Information Engineering, North China Institute of Aerospace Engineering, Langfang 065000, China; liu_ws0915@126.com (W.L.); d_jp424@163.com (J.D.); clx20010905@163.com (L.C.); teng_f2024@163.com (T.F.); 2 Hebei Collaborative Innovation Center for Aerospace Remote Sensing Information Processing and Application, Langfang 065000, China; 3 Xinjiang University of Science & Technology, Korla 841000, China; bhhtwh@126.com

**Keywords:** YOLOv7, aircraft recognition, SAR, Involution, attention mechanism

## Abstract

Synthetic aperture radar (SAR), as an active microwave imaging system, has the capability of all-weather and all-time observation. In response to the challenges of aircraft detection in SAR images due to the complex background interference caused by the continuous scattering of airport buildings and the demand for real-time processing, this paper proposes a YOLOv7-MTI recognition model that combines the attention mechanism and involution. By integrating the MTCN module and involution, performance is enhanced. The Multi-TASP-Conv network (MTCN) module aims to effectively extract low-level semantic and spatial information using a shared lightweight attention gate structure to achieve cross-dimensional interaction between “channels and space” with very few parameters, capturing the dependencies among multiple dimensions and improving feature representation ability. Involution helps the model adaptively adjust the weights of spatial positions through dynamic parameterized convolution kernels, strengthening the discrete strong scattering points specific to aircraft and suppressing the continuous scattering of the background, thereby alleviating the interference of complex backgrounds. Experiments on the SAR-AIRcraft-1.0 dataset, which includes seven categories such as A220, A320/321, A330, ARJ21, Boeing737, Boeing787, and others, show that the mAP and mRecall of YOLOv7-MTI reach 93.51% and 96.45%, respectively, outperforming Faster R-CNN, SSD, YOLOv5, YOLOv7, and YOLOv8. Compared with the basic YOLOv7, mAP is improved by 1.47%, mRecall by 1.64%, and FPS by 8.27%, achieving an effective balance between accuracy and speed, providing research ideas for SAR aircraft recognition.

## 1. Introduction

Synthetic aperture radar (SAR) [1] is an active microwave remote sensing technology, which has the capability of all-weather and all-time earth observation, greatly expanding the temporal and spatial scope of remote sensing observation [2,3]. Aircraft, as typical artificial targets, are numerous and diverse in type, presenting significant potential for observation [4]. Target recognition methods based on SAR images can obtain the type, quantity, and distribution location of aircraft [5], which can improve the efficiency and safety of air transportation in aspects such as aircraft scheduling and airport management. However, unlike traditional optical CCD imaging that forms a two-dimensional image at once [6], the two dimensions of SAR imaging are separate, namely range information and azimuth information perpendicular to the range. The imaging results are affected by the type of sensor and the observation direction, resulting in significant differences in the images of the same target under different conditions [7]. At the same time, during the SAR imaging process, buildings in an airport or some metal facilities around the aircraft can easily cause strong scattering similar to aircraft targets. These complex background clutters cause visual confusion to a certain extent, presenting the characteristic of “seeing is not knowing”, making it difficult to accurately locate and identify the target [8]. Moreover, in practical application scenarios, the recognition efficiency of the model also needs to be taken into account to ensure it can meet the requirements of real-time processing.

Traditional SAR image target recognition methods mainly rely on manual feature extraction and classifier design, but these methods require prior information and lack robustness. The representative algorithm is the constant false alarm rate (CFAR) algorithm [9]. CFAR utilizes the feature of radar cross section (RCS) to adaptively set the detection threshold and performs pixel-by-pixel detection on SAR images based on a local sliding window. Each pixel in the image participates in the sliding window operation multiple times, resulting in a generally low computational speed of the algorithm. Its derivative algorithms include CA-CFAR [10], VI-CFAR [11], OS-CFAR [12], SO-CFAR [13], and GO-CFAR [12]. CA-CFAR estimates the background noise by calculating the mean of the local area and is suitable for uniform backgrounds; OS-CFAR selects the median of the pixel values for noise estimation and is more suitable for small target detection, while GO-CFAR and SO-CFAR are, respectively, optimized for clutter edges and closely spaced targets. However, the CFAR algorithm only detects based on the contrast of RCS features and fails to effectively utilize the structural feature information of the target, affecting the precise localization of the detected target. Moreover, in practical application scenarios, the prior information of the target is often difficult to obtain in advance, and the algorithm’s computational efficiency is low, making it difficult to meet the requirements of real-time processing. In recent years, the rapid development of deep learning theory and technology has brought significant progress to the field of SAR image target recognition. Compared with traditional SAR image target detection methods, methods based on convolutional neural networks (CNNs) can achieve higher detection accuracy and faster detection speed due to their “end-to-end” detection characteristics [14]. For example, these include two-stage algorithms such as Faster R-CNN and one-stage algorithms such as SSD, YOLOv5, and YOLOv7. Compared with the two-stage algorithms that first partition and then classify, one-stage algorithms directly perform classification and regression predictions, have a simpler algorithm structure, and a faster training speed, thus having greater application potential in real-time scenarios.

Zhang et al. [15] presented a “three-look network” based on Faster R-CNN, achieving an “airport detection–runway extraction–aircraft detection” three-view design, which narrowed the detection range and achieved an F1 value of 0.67. Wang et al. [16] utilized an airport detection algorithm to generate runway masks, combined with a weighted bidirectional feature pyramid network (BiFPN) and adaptive spatial feature fusion (ASFF) to enhance the saliency of aircraft targets, achieving a detection accuracy of 95.4%. However, this two-stage strategy is not an end-to-end design, and the detection process is relatively cumbersome. Moreover, as a post-processing technique for eliminating false alarms [17], the extraction accuracy and timeliness of airport masks may also directly affect the overall efficiency and accuracy of the algorithm. Additionally, the combination of multi-scale feature fusion and visual attention mechanisms is a popular research direction in the field of target detection [18]. Current research mainly focuses on designing efficient multi-scale fusion modules to promote cross-scale information exchange, integrate high- and low-level information, and enhance multi-scale prediction to improve the adaptability of the algorithm to aircraft targets of different scales in SAR images [19,20,21]. The visual attention mechanism simulates human brain vision and can adaptively focus on target regions [22], accurately capture features, and suppress background interference. Due to its small parameter and computational requirements, its introduction has a relatively small impact on the network’s operational efficiency. Zhao et al. [23] proposed a novel network of pyramid attention expansion, which starting from the direction of attention mechanisms and multi-scale fusion, enhanced the extraction and representation of semantic features of aircraft targets through the MBDCM module and CBAM module, achieving good results in SAR image aircraft target detection. Li et al. [24] proposed an ICB module that combines scattering mechanism characteristics to alleviate the interference of strong scattering points around aircraft by extracting gray features and enhancing spatial information to suppress interference and redundant information in complex environments. Zhao et al. [25] addressed the issue of weak and shallow semantic information feature representation of aircraft by introducing a bottleneck attention module (BAM) and constructing an attention bidirectional feature fusion module (ABFFM), improving the algorithm’s extraction of shallow semantic features of an aircraft’s discrete scattering points. Luo et al. [26], based on YOLOv5s, combined an effective residual random attention module to highlight aircraft target features and overcome the interference of complex backgrounds and speckle noise, achieving a detection accuracy of 93.05%.

In order to quickly detect and accurately identify aircraft targets in complex backgrounds, an end-to-end SAR aircraft target recognition model, YOLOv7-MTI, is designed in this study. Firstly, the MTCN feature enhancement module is introduced into the backbone deep network to enhance the discriminative features and recognition speed of aircraft. Secondly, involution is introduced into the neck network to strengthen the discrete strong scattering points unique to the aircraft and suppress continuous scattering in the background. Experimental results show that the mean average precision (mAP) of the proposed network reaches 93.51%, which is 1.47% higher than that of YOLOv7, which is better than other comparison networks.

## 2. Materials and Methods

### 2.1. Overall Detection Framework

**Figure 1:**
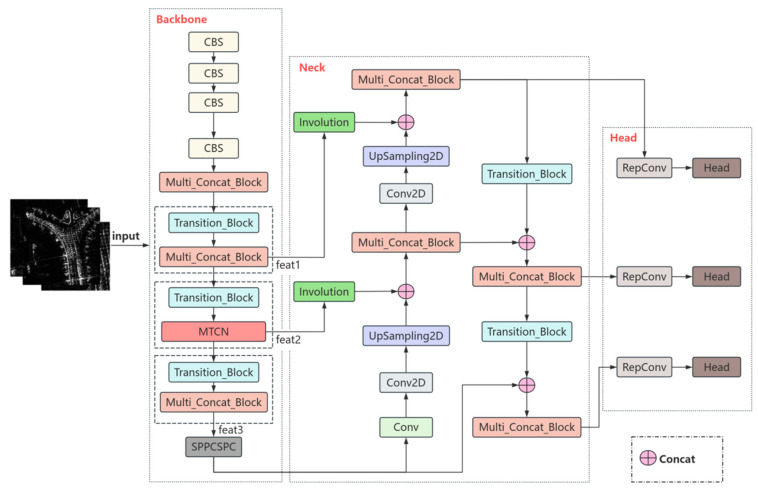
The framework of the YOLOv7-MTI model.

As shown in Figure 1, this study proposes a network, YOLOv7-MTI, which combines an attention mechanism and inner convolution operator for automatic aircraft recognition in complex scenes of SAR images. YOLOv7 is selected as the basic network, and MTCN is selected in the feat2 part of the backbone network to enrich the expression of features. MTCN is an object detection module composed of three triplet attention stacked PConv (TASP-Conv) units. In addition, involution is used to realize feature conversion from the backbone network to the neck network, which retains more spatial information while reducing the number of channels and suppresses the continuous scattering of the background. YOLOv7-MTI performs a resize on the input data to 640 × 640, and then the backbone network extracts SAR features to generate multi-layer feature maps. The three-dimensional tensor is denoted as X∈RC*H*W, where C, H, and W represent the channel size, height, and width of the feature map, respectively. The neck network integrates the feature maps of different scales and transmits them to the detection head. The detection head further refines and enhances the feature map.

### 2.2. MTCN Module

In the backbone network, $feat1\in R^{512*80*80}$ is a low-level feature map that provides rich spatial details of the object, which is helpful for object localization. $feat3\in R^{1024*20*20}$ is a high-level feature map in which $feat2\in R^{1024*40*40}$ encodes abstract semantics for distinguishing the foreground and background.

Limited by the imaging characteristics of SAR, the representation of aircraft is in a discrete state, and the structural and geometric features are not obvious [27]. However, the downsampling process of the backbone network will lead to an unnecessary loss of these aircraft features, which will affect the detection and recognition accuracy of the network. At the same time, we must consider that the aircraft features in SAR images are mainly geometric shapes, such as the aircraft aspect ratio. Taking into account spatial relationships, such as the relative position of the wing and tail and other low-level semantic information [20], this study designs the MTCN module to extract low-level features.

**Figure 2:**
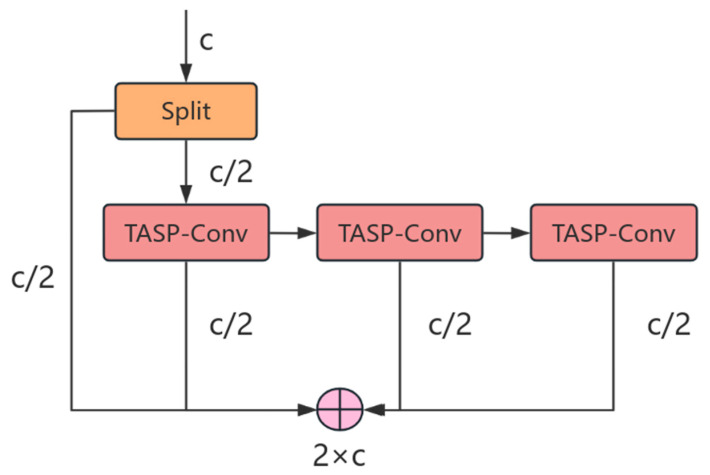
The structure of the MTCN.

The structure of the MTCN module is shown in Figure 2, which is mainly composed of TASP-Conv modules. The input features are divided into two parts by the split operation. One part retains the original features to avoid information loss, and the other part enters the TASP-Conv module for further feature extraction. Considering the multi-scale characteristics of aircraft, a three-layer TASP-Conv module is used to cover the semantic granularity from coarse to fine.

**Figure 3:**
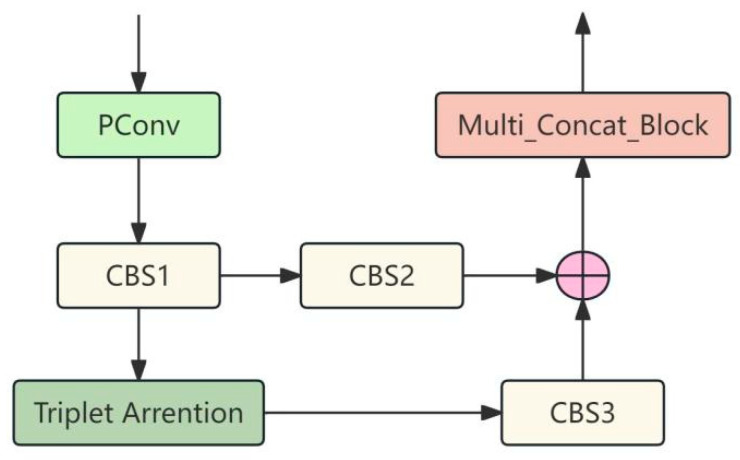
The structure of TASP-Conv.

The TASP-Conv module is shown in Figure 3. In order to not increase the number of calculations, the input features are subjected to PConv convolution operation, and then the number of channels is expanded through CBS1 to maintain the feature diversity of the incoming target attention module (TAM) [28] and achieve lower delay. The features processed by the TAM are adjusted back to the original number of channels by CBS3 and connected with the features adjusted by CBS2 through the Concat channel before being passed to Multi_Concat_Block for multi-scale feature extraction.

**Figure 4:**
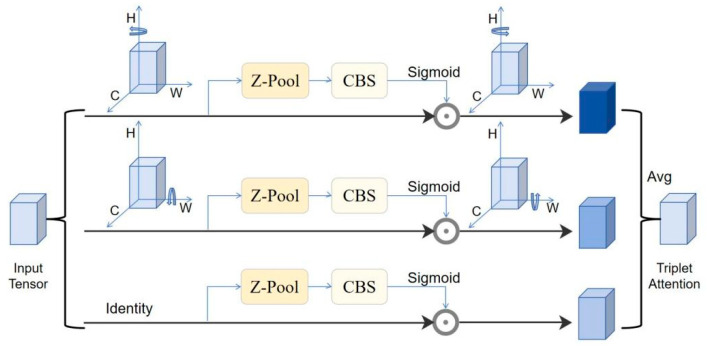
The Structure of the TAM.

When the traditional network performs downsampling, the pixel information of the target will be significantly lost, which is not obvious in the optical image. However, in the SAR image aircraft recognition task, due to the discrete type of aircraft image, the assumption of redundant information is no longer valid. In 2020, Diganta [28] proposed a (TAM), whose network structure is shown in Figure 4. This attention mechanism adopts a three-branch parallel structure to model the feature interaction relationships of channel–height, channel–width, and height–width, respectively. Through ablation experiments in different networks, the researchers verified the effectiveness of the TAM. The first two branches of the TAM achieve feature dimension reduction by applying Z-pooling after rotating the input tensor and then generating attention weights through k × k convolution, batch normalization, and sigmoid activation function; the third branch directly models the dependency relationship between spatial dimensions. In this study, we utilize the multi-dimensional feature interaction mechanism of the TAM to effectively enhance the model’s ability to model global context information while maintaining a relatively low computational cost.

**Figure 5:**
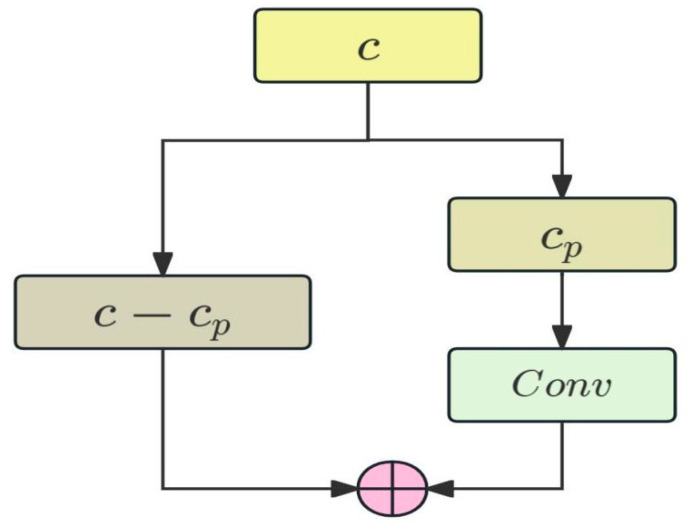
The structure of PConv.

To achieve faster neural networks, Chen et al. [29] proposed the PConv structure (Figure 5), which more effectively extracts spatial features by simultaneously reducing redundant computations and memory accesses. PConv only performs 3 × 3 convolution operations on the $c_{p}$ channels passed in, while the remaining $c-c_{p}$ channels remain unchanged, significantly reducing the computational load. Since feat2 is a mid-level feature map, it is used to balance semantic and detailed information. The TAM extracts detailed information, and PConv selects the first half of the channel for convolution operation and retains the high-level semantic features in the second half of the channel, which helps to maintain the representation ability of the network.

### 2.3. Involution

**Figure 6:**
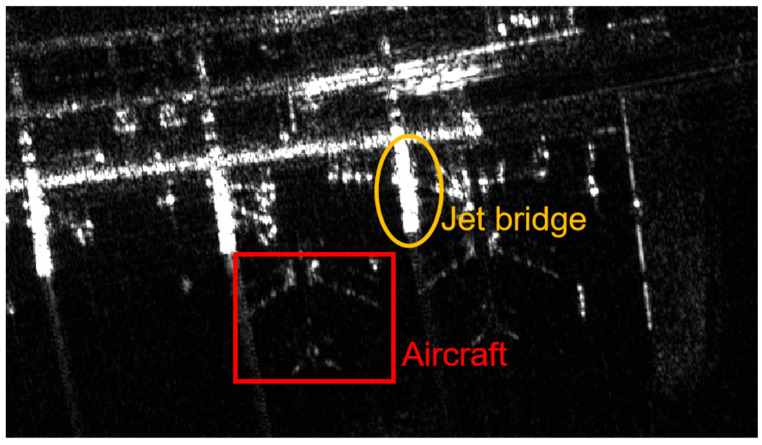
Aircraft docking environment.

As shown in Figure 6, the highly reflective strong echoes from metal facilities such as the jet bridge can form bright areas with strong scattering similar to aircraft targets. Meanwhile, the aircraft targets parked near the jet bridge appear relatively dim in the image. This complex electromagnetic scattering environment can severely interfere with the SAR echo signals of aircraft targets, increasing the difficulty of acquiring a precise target location.

Traditional Conv operations have two main characteristics, namely spatial-agnostic and channel-specific [30]. In the spatial domain, convolutional kernels ensure efficiency by being reused at different positions and pursue translation equivariance. In the channel domain, multiple convolutional kernels are responsible for capturing various information encoded in different channels to meet channel specificity. Since VGGNet [31], modern neural networks typically limit the spatial span of convolutional kernels to within 3 × 3 to satisfy the compactness of the convolutional kernels. However, traditional convolutional kernels cannot adapt to visual patterns at different spatial positions and cannot adjust their weights according to different positions, which is not conducive to processing SAR aircraft targets in complex backgrounds.

Inspired by Li [32], involution uses a dynamic convolution kernel to adaptively assign weights at different positions so as to prioritize the most informative visual elements in the spatial domain and highlight the aircraft target. Involution reverses the two design principles of conventional convolution kernels from “spatial independence, frequency domain specificity” to “spatial specificity, frequency domain independence”, that is, the feature maps in each group share the parameters of a convolution kernel, but different convolution kernels are used for different spatial positions in the same group. This dynamic parameterized convolution kernel can enhance the discrete strong scattering points of aircraft, suppress the continuous scattering of background, and alleviate the interference of complex backgrounds.

**Figure 7:**
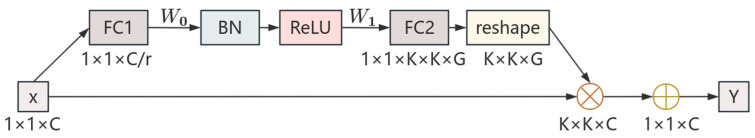
The structure of the involution.

As shown in Figure 7, involution uncoils the feature vector at one coordinate of the input feature into the shape of a kernel by Φ(FC-BN-ReLU-FC) and reshape (channel-to-space) transformations. To obtain the coordinates of points on the corresponding involution kernel, set the input feature neighborhood of the coordinate points on the feature vector to Multiply–Add to obtain the final output features. The Φ(FC-BN-ReLU-FC) operation is written as

(1) $$H_{i,j}=\Phi \left(X_{i,j}\right)=W_{1}\sigma \left(W_{0}X_{i,j}\right)$$

In the formula, $X_{i,j}$ represents a single pixel at coordinates (i,j) on the input feature map, $W_{0}\in R^{C/r*C}$ and $W_{1}\in R^{(K*K*G)*C/r}$ are linear transformation matrices, G denotes the number of groups, r is the channel reduction ratio which controls the scaling factor to adjust parameter quantity, and σ represents the intermediate BN and ReLU operations.

## 3. Experimental Results and Analysis

### 3.1. Aircraft Recognition Dataset

The experimental data utilized the SAR-AIRcraft-1.0 dataset [33], made open source in 2023. The images in this dataset are from the Gaofen-3 satellite and contain 16,463 aircraft target instances covering seven categories, including A220, A320/321, A330, ARJ21, Boeing737, Boeing787, and others. The distribution of the number of aircraft in each category within the dataset is shown in Table 1.

### 3.2. Evaluation Metrics

This research use precision, recall, F1 score, average precision (AP), and the mAP evaluation of aircraft recognition accuracy; FPS was used to evaluate the detection rate of the model.

Recall (Equation (2)) measures how well a model can correctly identify all the actual positive examples in a recognition task, which is the ratio of the number of positive examples identified by the model to the number of positive examples that actually exist.

(2) Recall=TP/(TP+FN)

Take the A220 class as an example, where TP is the fraction predicted by the model as A220 and also actually as A220; FP is the part of the model that incorrectly identifies the other class as A220. FN is the part of the model that incorrectly identifies A220 as another aircraft type.

Precision (Equation (3)) reflects the reliability of the model in the prediction of positive samples, that is, the proportion of samples that the model identifies as positive samples that are actually positive samples.

(3) Precision=TP/(TP+FP)

The F1 score (Equation (4)) intuitively reflects a trade-off effect of the model in accurately identifying positive samples and avoiding false positives. It is used to measure the harmonic mean of precision and recall at the same time due to improper data division.

(4) F1=2×(Precision×Recall)/(Precision+Recall)

AP (Equation (5)) is the area under the precision–recall curve.

(5) $$AP=\int_{0}^{1}Precision\left(Recall\right)dR$$

mAP (Equation (6)) is the average of the average detection accuracies across all classes.

(6) $$mAP=\sum_{i=1}^{n}AP\left(i\right)/n$$

FPS (Equation (7)) is a measure of the speed of image processing or model inference and represents the number of image frames processed in one second.

(7) FPS=1/Latency

This is how long it takes to process a single frame.

### 3.3. Implementation Details

The system environment of this study is Windows 11 with a graphics card model of NVIDIA GeForce RTX 4050 (6 GB) and a CPU of 13th Gen Intel^®^ Core™ i5-13500H (16 cores). The training and testing environment was built based on the CUDA 11.3 and PyTorch 1.11.0 frameworks. During the training process, the training parameters set included epoch (number of iterations), batch size (number of images in each batch), lr0 (initial learning rate), lr1 (final learning rate), momentum (momentum parameter), etc. Among them, epoch was set to 100, batch size to 2, lr0 to 1 × 10^−3^, lr1 to 1 × 10^−3^ × 0.01, and momentum to 0.937 [24]. The IoU threshold was set to 0.5, and the Adam optimizer was used to iteratively update the network model parameters. The input image size was uniformly scaled to 640 × 640.

### 3.4. Ablation Experiment

In order to verify the promotion effect of the TAM module, PConv module, MTCN module, and involution on the YOLOv7 network, a series of ablation experiments were designed. For fair comparison, the same hyperparameter settings were maintained in all ablation experiments. The results of ablation experiments for each module are shown in Table 2 and Table 3. PConv (F) represents the convolution calculation of the first half of the channel in PConv, and PConv (L) represents the convolution calculation of the second half of the channel in PConv.

From the experimental results in the table, it can be seen that the AP, mAP, mPrecision, and FPS of aircraft categories except A320/321 have been improved after the introduction of the TAM. In the SAR-AIRcraft-1.0 dataset, A320 and A321 are classified into the same category, but there are slight differences between the two types of aircraft. Although the shared lightweight attention gate structure adopted by the TAM can effectively amplify the salient features, this design has certain limitations: when the features of some A320/A321 samples deviate from the standard salient features, the model may not accurately identify them. This is proved by the decrease in AP and model mRecall for A320/321. Table 3 shows that PConv has the highest FPS, while PConv (F) has a better mAP than PConv (L), which verifies the rationality of preserving semantic features in deep networks. However, the sparse computing characteristics of PConv will exacerbate the impact of insufficient data [34], and its local convolution mechanism is highly dependent on data distribution, which makes it difficult to accurately capture the key local features of A330.

**Figure 8:**
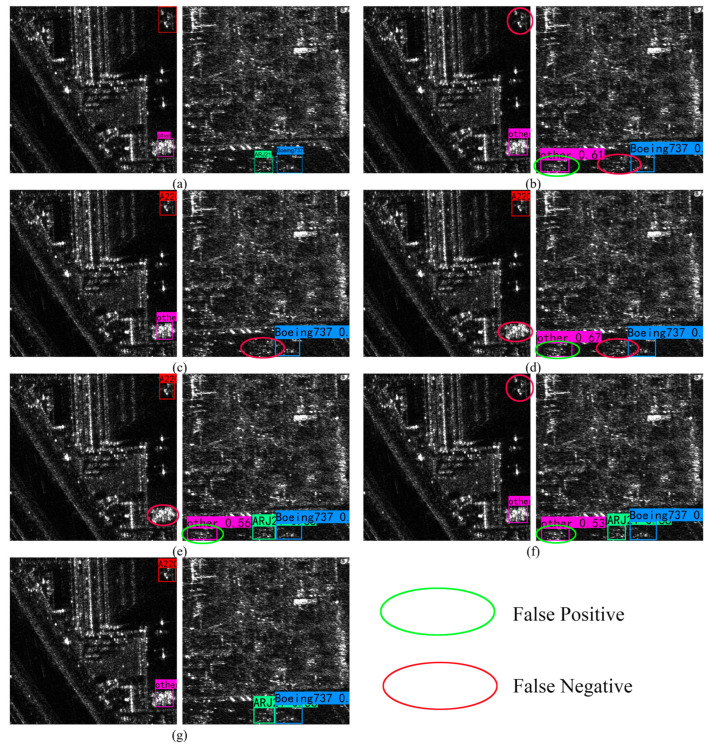
Results of ablation experiment identification. The green circles indicate the false detection areas, and the red circles indicate the missed detection areas. (**a**) Label; (**b**) baseline YOLOv7; (**c**) + TAM; (**d**) + PConv (F); (**e**) + MTCN; (**f**) + involution; (**g**) YOLOv7-MTI.

After adopting the MTCN module, the network improves the mAP, mPrecision and mF1. To further verify the effectiveness of the MTCN module, this study shows the recognition results of each module, as shown in Figure 8. Figure 8e shows the recognition results of + MTCN, which recognizes the missed weak scattering targets in Figure 8c,d and has high sensitivity to the weak scattering aircraft targets in SAR images; however, it misses the strong scattering targets. Figure 8f shows the recognition result of + involution, which can be seen to respond significantly to strong scattering aircraft targets in SAR images. When only involution is used in the neck part, the AP of the ARJ21 category reaches 96.81%. This is because ARJ21 shows the characteristics of short fuselage with strong scattering, and involution will dynamically generate compact nuclei to focus on the middle of the fuselage and highlight the target features. Compared with other modules, the mRecall of involution is improved to 96.08%, indicating the effectiveness of involution in highlighting aircraft features and alleviating complex background interference, and more aircraft targets can be detected.

**Figure 9:**
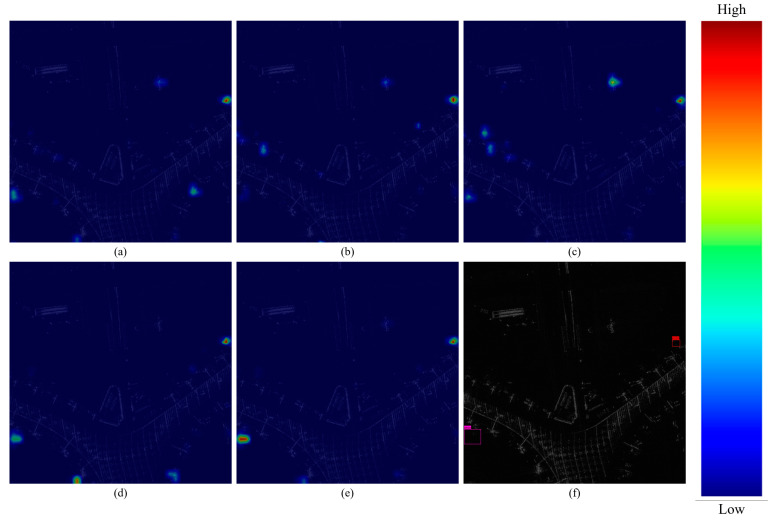
Heat map visualization. (**a**) + TAM; (**b**) + PConv (F); (**c**) + MTCN; (**d**) + involution; (**e**) YOLOv7-MTI; (**f**) label.

In order to intuitively observe the ability of different modules to extract network features, Figure 9 shows the feature map visualization results of each module. It can be seen that the MTCN module is easily affected by complex background clutter and easy-to-detect multi-targets, resulting in a large probability of false detection. Involution can suppress the linear scattering of corridor bridges, but due to the lack of channel information interaction, it mistakenly focuses on discrete feature regions like aircraft. YOLOv7-MTI integrates the advantages of both; the MTCN module increases the channel–space interaction, and involution prioritizes visual elements to suppress background clutter and complete accurate feature extraction.

In the aircraft target recognition task, it is necessary to balance detection accuracy and real-time performance to achieve efficient recognition. The MTCN module based on the TAM and PConv improves the detection rate from 16.82 FPS of baseline YOLOv7 to 25.46FPS (an increase of 51.4%), while YOLOv7-MTI with further involution has a slight speed loss (25.09FPS). It still meets the real-time detection requirements of ≥25FPS [35].

### 3.5. Network Comparison Experiment

In order to further verify the effectiveness of this study, four representative CNN-based detection networks were selected for comparison, including the accuracy evaluation results of mAP, mPrecision, mRecall, and mF1 of Fast-RCNN, SSD, YOLOv5 and YOLOv8 (Table 4).

The mAP, mPrecision, mRecall, and mF1 of YOLOv7-MTI are 93.51%, 81.42%, 96.45%, and 88.1%, respectively, and the aircraft target recognition accuracy is the highest. The recognition accuracy of the Faster R-CNN network is the lowest. Because the mechanism designed by the two-stage algorithm based on optical image features relies on selective search and RPN to generate candidate regions, the optical image target features are clear, the SAR image is disturbed by noise, and the edge is blurred and the scattering is variable. As a result, it is easy to miss detection or generate invalid aircraft target candidate regions in SAR images. In contrast, single-stage detection algorithms such as SSD, YOLOv5, and YOLOv8 are more suitable for SAR aircraft target recognition tasks. Although SSD has a certain ability to identify SAR aircraft targets, demonstrating the highest mPrecision, the mRecall is only 43.31%, indicating that more than half of the real targets are not detected. The overall performance of YOLOv5 is between SSD and YOLOv7-MTI. Although YOLOv5 can achieve a certain degree of aircraft recognition, it has low accuracy when dealing with weak-signal aircraft in complex environments due to insufficient target positioning ability, which affects the recognition effect. Although the recognition accuracy of YOLOv8 reaches 93.43%, the mRecall is much lower than that of YOLOv7-MTI. Considering the particularity of the aircraft target and following the safety logic of “it is better to misreport one hundred times than miss one” in the aviation field, the network with the higher mRecall should be selected.

**Figure 10:**
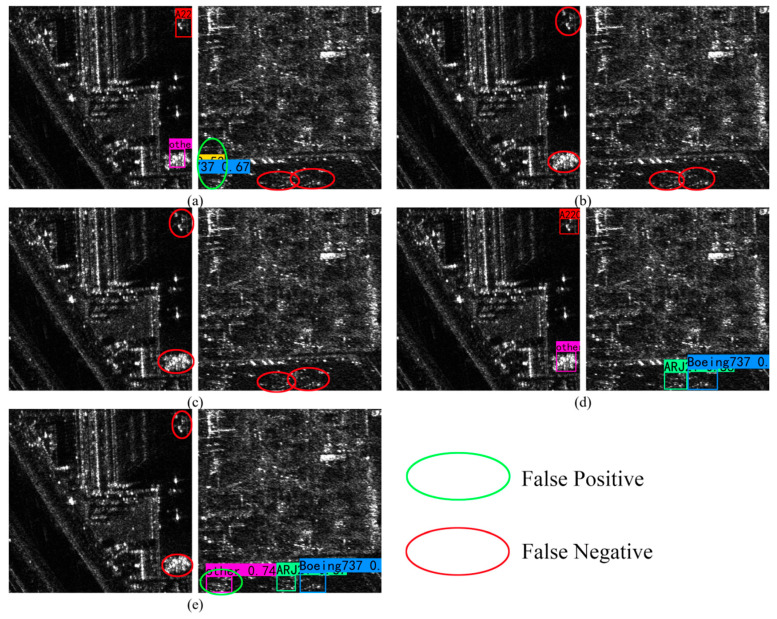
Results of ablation comparative identification. The green circle represents the false detection area, and the red circle represents the missed detection area. (**a**) Faster R-CNN; (**b**) SSD; (**c**) YOLOv5; (**d**) YOLOv7-MTI; (**e**) YOLOv8.

Figure 10 shows the aircraft recognition results of five kinds of networks in complex and large-scene SAR images. It can be seen that the SSD and YOLOv5 networks fail in this scene and fail to detect any aircraft targets, which seriously lacks the ability to locate targets in complex backgrounds. Due to the limitations of the fixed default box design and the multi-scale feature fusion mechanism, it is difficult to adapt the SSD network to the size change in the target in SAR images. However, the FPN + PAN feature pyramid structure adopted by the YOLOv5 network has a small number of feature fusion layers, which insufficiently captures the context information of small targets. Although the Faster R-CNN algorithm can detect parts of aircraft targets, due to the loss of small target feature information caused by RoI pooling operation in its two-stage detection process, there are serious missed detection problems in the detection results and a large number of invalid candidate regions. YOLOv8 can detect some targets, but it is not sensitive to strong scattering targets. In contrast, the YOLOv7-MTI network can accurately locate the position of the aircraft, extract the features of different types of aircraft, and achieve competitive detection performance.

### 3.6. Comparison Test of MTCN

In order to verify the effectiveness of the MTCN module proposed in this study, the module insertion was performed on the feat2 positions of YOLOv5, YOLOv7, and YOLOv8. The results are shown in Table 5. After using the MTCN module, the mAP of YOLOv5, YOLOv7, and YOLOv8 is increased by 4.5%, 0.99%, and 1.1%, and the mPrecision is increased by 0.88%, 0.81%, and 1.27%, respectively. The results show that the MTCN module has a positive effect on the YOLO network. It further proves the rationality of the module design.

## 4. Discussion

At present, most of the mainstream target recognition networks are designed for optical images, and the research on SAR images is relatively immature. In this study, an effective aircraft recognition network for SAR images is proposed to improve the accuracy and recognition speed of aircraft recognition.

In the selection of the base network, the trade-off between accuracy and speed is considered, and the YOLO series meets this requirement. The risk of missing an aircraft is much higher than that of false detection. Therefore, according to the experimental results in Table 4, this study chooses YOLOv7 with a higher mRecall as the basic network. According to the results in Table 2 and Table 3, the recognition speed of YOLOv7 is less than 25FPS. The deep design of the MTCN module allows the overall performance of the model to achieve significant improvement, reaching 93.03% mAP, but when affected by the TAM, the mRecall is the lowest. The mRecall of involution is 96.08%, which can alleviate the continuous scattering of the background and detect most of the aircraft, but it has a negative effect on Boeing787 and A220 aircraft. Compared with YOLOv7, the AP floating of seven aircraft models is controlled between −0.34% to 2.75, and the FPS reaches 25.09, which achieves a good balance between recognition accuracy and speed in complex scenes.

## 5. Conclusions

Aircraft target recognition technology in SAR images is of great significance to improve the ability of civil aviation supervision. The ability to accurately detect and identify aircraft targets plays a key role in airspace security control. This study focuses on the collaborative optimization problem of fast detection and accurate recognition in SAR image aircraft recognition under complex backgrounds and proposes a YOLOv7-MTI network. The performance improvement of YOLOv7-MTI mainly comes from two modules, the MTCN module and involution working in series, which effectively integrates the “channel space” interaction information of the aircraft and improves the recognition accuracy and efficiency. Experimental results on the SAR-airplane-1.0 dataset show that YOLOv7-MTI can detect more AIRcraft targets, suppress the interference of complex backgrounds, and enhance real-time expression.

However, the noise in SAR images often seriously covers the shape contour and texture features of the target. How to dynamically remove multiplicative noise and retain the structural features of aircraft signals will be a significant research hotspot.

## Figures and Tables

**Table 1:** Number of categories in the SAR-AIRcraft-1.0 dataset.

Categories	Boeing787	Boeing737	A220	A330	other	ARJ21	A320or321
Number	2645	2557	3730	309	4264	1187	1771

**Table 2:** Ablation experiment AP table.

	A330	Boeing787	Boeing737	ARJ21	A320/321	A220	Other
YOLOv7	97.92	95.42	93.44	91.11	89.20	88.99	88.20
TAM	99.36	95.67	93.65	93.81	88.23	89.87	89.09
PConv(F)	97.32	96.10	93.82	95.70	88.75	88.71	90.55
PConv(L)	97.23	95.34	94.67	92.87	88.84	87.54	90.65
MTCN	98.79	95.80	93.31	92.64	89.44	89.83	91.00
Involution	99.20	94.74	93.39	96.81	89.51	88.42	91.81
YOLOv7-MTI	99.43	96.37	93.10	93.86	91.26	90.40	90.12

Blue indicates the lowest value in this column, red indicates the highest value in this column, and the score is in percent system.

**Table 4:** Average indexes of comparative experiments.

	Faster R-CNN	SSD	YOLOv5	YOLOv8	YOLOv7-MTI
mPrecision	49.35	75.42	88.10	93.43	93.51
mRecall	47.97	93.00	87.14	86.76	81.42
mF1	56.56	43.31	79.47	87.03	96.45
FPS	50.14	57.43	82.86	86.71	88.10

Blue indicates the minimum value of each row and red indicates the maximum value. The scoring system is out of 100.

**Table 5:** Comparative experimental results.

	mAP	mAP (MTCN)	mPrecision	mPrecision (MTCN)	mRecall	mRecall (MTCN)
YOLOv5	88.10	92.60	87.14	88.02	79.47	86.56
YOLOv7	92.04	93.03	82.67	83.48	94.81	94.42
YOLOv8	93.43	94.53	86.76	88.03	87.03	86.56

**Table 3:** Average indexes of ablation experiments.

	YOLOv7	TAM	PConv(F)	PConv(L)	MTCN	Involution	YOLOv7-MTI
mAP	92.04	92.81	92.99	92.45	93.03	93.41	93.51
mPrecision	82.67	83.33	82.65	82.69	83.48	82.16	81.42
mRecall	94.81	93.52	94.83	94.91	94.42	96.08	96.45
mF1	88.33	88.12	88.29	88.28	88.71	88.42	88.29
FPS	16.82	25.40	25.75	25.70	25.46	24.28	25.09

Blue indicates the minimum value of the line, red indicates the maximum value of the line, and the score is in a percent system.

## Data Availability

The SAR-AIRcraft-1.0 dataset is available for download at: https://radars.ac.cn/web/data/getData?newsColumnId=f896637b-af23-4209-8bcc-9320fceaba19.
